# Protein-Tyrosine Kinase Signaling in the Biological Functions Associated with Sperm

**DOI:** 10.1155/2012/181560

**Published:** 2012-11-11

**Authors:** Takashi W. Ijiri, A. K. M. Mahbub Hasan, Ken-ichi Sato

**Affiliations:** ^1^Laboratory of Cell Signaling and Development, Department of Molecular Biosciences, Faculty of Life Sciences, Kyoto Sangyo University, Kyoto 603-8555, Japan; ^2^Laboratory of Gene Biology, Department of Biochemistry and Molecular Biology, University of Dhaka, Dhaka 1000, Bangladesh

## Abstract

In sexual reproduction, two gamete cells (i.e., egg and sperm) fuse (fertilization) to create a newborn with a genetic identity distinct from those of the parents. In the course of these developmental processes, a variety of signal transduction events occur simultaneously in each of the two gametes, as well as in the fertilized egg/zygote/early embryo. In particular, a growing body of knowledge suggests that the tyrosine kinase Src and/or other protein-tyrosine kinases are important elements that facilitate successful implementation of the aforementioned processes in many animal species. In this paper, we summarize recent findings on the roles of protein-tyrosine phosphorylation in many sperm-related processes (from spermatogenesis to epididymal maturation, capacitation, acrosomal exocytosis, and fertilization).

## 1. Introduction

Protein-tyrosine kinase (PTK) activity and tyrosine phosphorylation of cellular protein were initially discovered by Hunter and colleagues [1–3]; they analyzed the protein kinase activity associated with the protein complex of polyoma virus middle T antigen and viral Src gene product, a cellular counterpart of which is the cellular Src protein. At that time, phosphorylation events on amino acids other than tyrosine (i.e., serine and threonine residues) were already known as posttranslational modifications of physiological importance. However, the discovery of tyrosine phosphorylation for the first time opened a window to understand the relationship between protein phosphorylation (including serine/threonine phosphorylation) and malignant cell transformation (e.g., development of cancer) [4]. In addition, a growing body of evidence has demonstrated that tyrosine phosphorylation catalyzed by cellular Src and other PTKs expressed in normal cells and tissues regulates a variety of cellular functions such as developmental processes, disorder of normal cell functions, immunological responses, neuronal differentiation and transmission, pathological infection, and senescence. Thus, protein-tyrosine phosphorylation has emerged as a signal transduction mechanism of fundamental importance in all eukaryotic cells and, in some cases, prokaryotic cell behavior [5–7]. 

**Figure 1:**
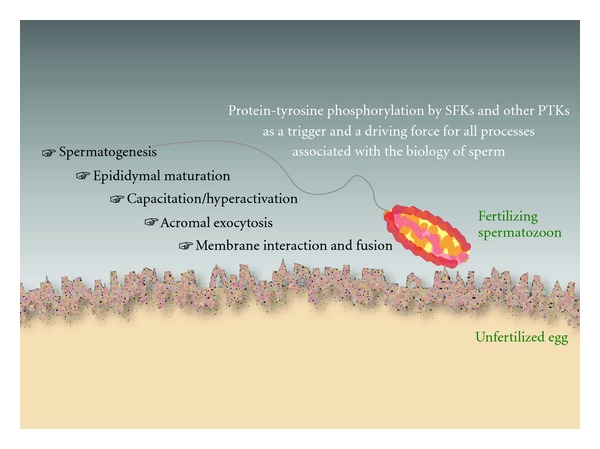
Protein-tyrosine phosphorylation and the biology of sperm. A sequence of events in the sperm must be done to facilitate a successful fertilization. The events include spermatogenesis and epididymal maturation that occur in the male reproductive organs, capacitation/hyperactivation and acrosomal exocytosis (or acrosome reaction, AE) in the female reproductive tract (in the case of species employing internal fertilization: e.g., mammals) or in the extracellular space (in the case of species employing external fertilization: e.g., frogs and fishes), and gamete interaction and fusion at the plasma membranes. In all of these processes, protein-tyrosine phosphorylation catalyzed by SFKs (e.g., Src) and/or other PTKs (e.g., EGFR, Abl) is suggested to play an important role. For details, see text.

 In the sexual reproduction system, two different kinds of gamete cell: egg and sperm, interact and fuse with each other to accomplish fertilization that gives rise to a newborn [8]. In this fundamental biological event, both egg and sperm undergo a number of biochemical and cell biological reactions that culminate in successful embryogenesis and early development. Especially in the case of multicellular organisms including humans, egg and sperm are special cells in view of their appearance as a single cell. To become such a specialized type of cell, the ancestor of the gametes, that is, primordial germ cell (PGC), along with sex determination in the host, must undergo meiotic cell division [9]. Moreover, to become fully competent for fertilization, egg and sperm must undergo a series of “differentiation” or “maturation” events [10–12]. During the past several decades, a number of studies have dealt with the cellular and molecular mechanisms of gametogenesis, fertilization, and embryogenesis. Among these are characterizations of protein-tyrosine phosphorylation in these events that involved identification of the responsible PTKs (e.g., Src), their regulators and substrates, and evaluation of their roles for cellular functions [13–19]. In this paper, we will briefly discuss the biology of sperm (gametogenesis, differentiation, maturation, and fertilization), recent achievements in understanding the involvement of PTKs and protein-tyrosine phosphorylation in the biology of sperm, and future directions for this research field (Figure 1). 

## 2. General View of Sperm Biology

Spermatogenesis is a highly specialized process of cellular differentiation in which diploid progenitor cells of the testis differentiate into haploid spermatozoa [20]. The entire process is divided into three sequential mitotic, meiotic, and postmeiotic stages. In the male meiotic stage, after PGCs migrate into the genital ridges, they become gonocytes and start differentiation into spermatogonia at the basement of seminiferous tubules. Some of them, spermatogonial stem cells (SSCs), also retain the ability for self-renewal [21]. Owing to the role of SSCs, sperm are produced continually (more than 50,000,000 a day in humans) almost throughout the lifetime. Meiosis is the event in which chromosome pairing and genetic recombination occur in the functional tetraploid pachytene spermatocytes [22]. In this process, the genes are shuffled between homologous chromosomes, which results in genetic diversity. This helps the species to survive through natural selection.

 Most of the components found in mature spermatozoa are primarily produced at the postmeiotic phase in mammals, and developing spermatids display a variety of morphological and biochemical changes [23]. Many of the organelles in spermatids are transformed into specific structures; the acrosome originates from Golgi body and the main part of the flagellum is composed of spindle-shaped body. The flagellum contains cytoskeletal components and signal transduction mediators. The fibrous sheath, a unique mammalian cytoskeletal structure surrounding the axoneme, serves as a scaffold for constituents of signaling cascades in the regulation of sperm motility [24]. The nucleus is also changed into the tightly compacted shape and size of sperm head by the sequential replacement of the histones with transition proteins and protamines [25].

 All stages in spermatogenesis are regulated by the stage-specific expression of a wide variety of genes. Other factors that influence spermatogenesis are the interactions between Sertoli cells and testosterone produced from Leydig cells [26]. In female animals, however, the limited numbers of oogonia differentiate, progress through the first meiotic prophase, and are arrested in the infant ovary. Then, with the onset of adolescence, they mature to second meiotic metaphase and are arrested again. Some of them are released by ovulation and complete meiosis by the entry of a sperm [27]. Similar criteria for oogenesis and oocyte maturation also apply in other kinds of vertebrates, including frog and fish [28, 29]. Testicular sperm look morphologically mature, but they are immotile. Therefore, after sperm leave the testis, they require a further maturation process to acquire the functions for fertilizing an oocyte during transmission through the epididymis [30].

 The sperm pass through the caput, corpus, and cauda epididymides sequentially. Then, they are stored at the cauda epididymis until ejaculation. While transiting through the epididymis, they undergo biochemical and physiological modifications, resulting in the acquisition of basal motility and the ability to fertilize an oocyte. These modifications include changes in the glycosylation of acrosomal proteins [31, 32] and in the lipid composition of sperm [33], as well as elevations of cyclic adenosine monophosphate (cAMP) [34, 35] and negative charge on the sperm surface [36]. The other difference between caput and cauda epididymal sperm is the pattern of protein tyrosine phosphorylation [37].

 Mammalian sperm need to change their status further to acquire the ability to become competent to bind and fuse with an oocyte after release into the female reproductive tract [38, 39]. This change is termed “capacitation” and confers hyperactivated motility (hyperactivation) and an ability to undergo acrosomal exocytosis (AE) [40] or acrosome reaction to the sperm [8]. To be capacitated, sperm require a period of incubation and interaction in the female reproductive tract. However, this can be induced in vitro in an appropriate experimental medium [40]. Capacitation promotes changes in cholesterol content, plasma membrane fluidity, and intracellular ion concentrations [42]. Another good hallmark for capacitation is an increase in protein tyrosine phosphorylation [43]. Chemotaxis is a phenomenon that guides cells to undergo correct movement toward or away from certain chemicals. This is also known to be important for sperm to interact with an oocyte in the female reproductive tract [44], maybe because sperm are extremely small compared with oocytes. Chemotaxis for sperm guidance was discovered first in marine invertebrate species [45], then in amphibians and mammals [46]. In eutherian mammals, AE releases proteolytic enzymes from the acrosome stored in sperm head [8]. It was believed that these enzymes assist in sperm penetration through the zona pellucida (ZP), the glycoprotein coating on the surface of oocytes, and fusion with an oocyte. However, a recent observation from in vitro fertilization suggests that most sperm undergo AE before contact with the ZP [47], showing the need to reconsider the timing and biological significance of AE during a series of sperm events.

 Fertilization-related phenomena include gamete interaction and fusion, egg activation, polyspermy block, and nuclear fusion, all of which culminates in initiation of embryonic development. To date, the sperm membrane protein Izumo1 [48] and the oocyte surface protein CD9 [49–51] are reported to be indispensable for the fusion between sperm and the oocyte plasma membrane in mouse. The gamete fusion triggers repeated increases (e.g., mouse) or a transient elevation (e.g., frog) in intracellular calcium ([Ca^2+^]_i_) in oocyte, so-called Ca^2+^ oscillation or Ca^2+^ wave, which serves as an initiator of egg activation [17, 52, 53]. It is still debatable how sperm can act as a trigger for egg activation [19]. One possibility is receptor-mediated activator, while another is diffusible activator, “sperm factor.” Recent findings suggest that inositol trisphosphate (IP_3_) acts as a second messenger for the Ca^2+^ release reactions and that the egg-associated Src-phospholipase C*γ* (PLC*γ*) (e.g., sea urchin, frog) [14, 16] or the sperm-derived components such as PLC*ζ* (e.g., mouse) [54] and citrate synthase (newt) [55] mediate the gamete interaction/fusion and the activation of IP_3_-dependent Ca^2+^ release. After a sperm enters an oocyte, the nucleus has to be decondensed as a pronucleus for nuclear fusion. Then, the fertilized egg starts DNA synthesis for the following early embryogenesis.

 Recently, using proteomics approaches, a number of sperm proteins in mouse and rat have been identified as those that are phosphorylated on tyrosine residues during epididymal maturation and capacitation [56–58]. Our group has also reported important roles of Src family PTKs (SFKs) in the sperm-induced egg activation during gamete interaction and fusion by using the African clawed frog, *Xenopus laevis*. The following sections are an overview of the recent progress to understand the correlations between PTKs and various sperm events.

## 3. Involvement of PTKs in Spermatogenesis

**Figure 2:**
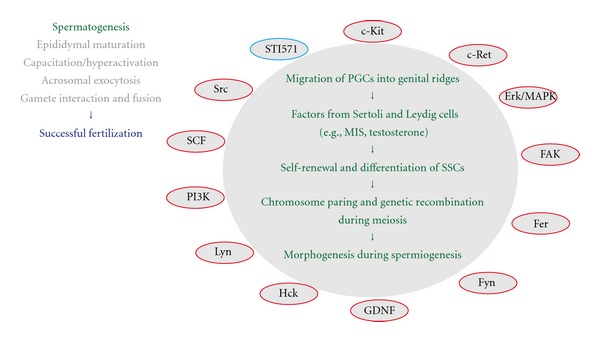
Protein-tyrosine phosphorylation and a sequence of events associated with spermatogenesis. For details of spermatogenesis and its associated signaling molecules (the full spelling of all abbreviations as well), see text. Note that MIS, Mullerian-inhibiting substance, is a testicular-differentiating factor that is produced in Sertoli cells and Leydig cells, whose differentiation is promoted by the actions of Sry and other sex-determining gene products. Also note that MIS acts in concert with testosterone. Note that positive regulators for protein-tyrosine phosphorylation are indicated in red circles

PTKs play various biological roles in many types of somatic cell, so it is not surprising that they act on spermatogenic cells and their supporting cells, that is, Sertoli cells, in the testis. Actually, it has been demonstrated that several families of PTKs including Src kinase are correlated with most spermatogenic events (Figure 2). In adult mouse testis, the protein expression of Src, Lyn, and Hck were observed, while the expressions of eight members of the SFK were detected by quantitative polymerase chain reaction. For instance, Src protein localizes weakly to the cytoplasm in spermatocytes and strongly in round and elongated spermatids, leading to strong accumulation in acrosome of cauda epididymal sperm with entire flagellum detection [59]. In humans, Src protein was detected strongly around the acrosomal region in round and elongated spermatids [60]. 

 c-Kit is a transmembrane tyrosine kinase receptor that binds to stem cell factor (SCF). SCF induces dimerization of c-Kit that activates the tyrosine kinase residues by autophosphorylation [61], leading to the downstream signaling through phospho-tyrosine-binding adaptor proteins such as PLC*γ*1. During mouse early development, c-Kit is essential for the migration of PGCs to the genital ridges in the embryo and then functions in the maintenance of PGCs [62]. Src kinase is also involved in this process [63]. In adult mouse testis, the expression of c-Kit is detected in differentiating spermatogonia, whereas the expression of SCF is detected in Sertoli cells; therefore, c-Kit/SCF is important for maintaining differentiating type A spermatogonia [64]. On the other hand, to maintain the property of self-renewal in mouse SSCs, c-Ret tyrosine kinase receptor mediates between glial cell line-derived neurotrophic factor (GDNF) and Src family kinase signaling [65]. Recently, it has also been suggested that c-Kit plays a pivotal role in regulating the ratio between differentiation and self-renewal during maintenance of the SSC population [66]. 

 There are a few reports about tyrosine phosphorylation in meiosis during spermatogenesis (Figure 2). Mouse has Fes-related proteins (Fer) of two different size [67], which correspond to 94 kDa or 51 kDa tyrosine kinase. The latter accumulates in primary spermatocytes where the cell cycle is in the first meiotic prophase, and the role of phosphorylation for the timing of meiosis entry is suggested in mammals as well as in yeast [68]. c-Kit/SCF system is also required for transition when mouse spermatogonia undergo cell division to enter meiosis [69]. This was also examined using a specific inhibitor to c-Kit (STI571), resulting in reduction of the number of mouse meiotic cells under the control of retinoic acid [70].

 Interestingly, some of the truncated forms of PTK seem to have roles in the process of sperm morphogenesis: spermiogenesis. At least three examples of nonreceptor tyrosine kinase, Fyn as well as Fer and Hck [71], have been reported. Truncated Fer was detected in the Golgi, acroplaxome, and manchette of rat spermatid [72], while truncated Hck was observed mainly at the acrosome of bovine sperm [73]. They are also suggested to regulate actin assembly via phosphorylation. Therefore, these observations suggest that truncated forms of Fer kinase and Hck may participate in sperm head shaping. Similarly, Golgi membrane in spermatids contains truncated Fyn that is missing the kinase domain, and this protein may be required for acrosome biogenesis [72]. A truncated isoform of c-Kit has also been detected in mouse round spermatids [74]. This protein lacks SCF-binding and dimerization domains, but retains a part of the kinase domain that would facilitate activation of PLC*γ*1 [75, 76]. It is suggested that truncated c-Kit is related to DNA integrity in human sperm [77]; however, its role is still unclear.

 Another correlation of PTKs with spermatogenesis is in the regulation of Sertoli cell tight junction, including at the blood-testis barrier (BTB). Male germ cells need to contact with Sertoli cells during most spermatogenic processes. Spermatogonia differentiate to preleptotene/leptotene spermatocytes in the basal compartment of the seminiferous epithelium. In addition, these spermatocytes have to translocate to the adluminal compartment of the seminiferous epithelium for further differentiation. However, there is the BTB, which acts as the immunological barrier between basal and adluminal compartments. Recently, it has been demonstrated that focal adhesion kinase (FAK), a nonreceptor tyrosine kinase, plays a key role in this process. FAK regulates the opening and/or closing of BTB by modulating the phosphorylation status of integral membrane proteins [78]. Besides, traditionally, FAK has been suggested to be involved in adherens junctions (AJ) between Sertoli and germ cells by the interactions with *β*1-integrin and other associated proteins including Src [78]. Moreover, Fer kinase has been shown to participate in the regulation of rat AJ [79]. Fyn-functions in the basal ectoplasmic specialization (ES) of actin filaments: at the junction between Sertoli cells as well as apical ES and at the junction between spermatids and Sertoli cells [80]. Apical ES also contains many lipids and protein kinases such as phosphatidylinositol 3-kinase (PI3K) and extracellular signal-regulated kinase/mitogen-activated protein kinase (Erk/MAPK), which are associated with Src [81].

 The major problem for research on mammalian spermatogenesis was the lack of a stable in vitro culture system, despite the efforts of many investigators [82, 83]. However, recently, an improved organ culture system using neonatal testis has been established, which can make SSCs differentiate to mature sperm in mouse [84]. This method for in vitro spermatogenesis should greatly facilitate the identification and characterization of more factors and genes correlated with PTKs for self-renewal and differentiation in spermatogonia, meiosis in spermatocytes, and morphogenesis in spermatids.

## 4. Involvement of PTKs in Epididymal Maturation

**Figure 3:**
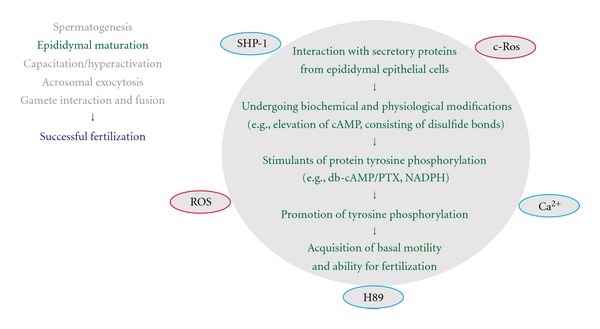
Protein-tyrosine phosphorylation and a sequence of events associated with epididymal maturation of sperm. For details of the epididymal maturation and its associated signaling molecules (the full spelling of all abbreviations as well), see text. Note that positive regulators for protein-tyrosine phosphorylation are indicated in red circles.

Like other cells, sperm need adenosine trisphosphate (ATP) as an energy resource for their functions, for example, motility. The dominant pathway for ATP production in mouse sperm is glycolysis, while spermatocytes and spermatids prefer oxidative phosphorylation [83–85]. It is suggested that this switching to glycolysis occurs during epididymal maturation in rabbit [86]. During epididymal maturation, sperm proteins contain a greater number of disulfide bonds, leading to the stabilization of sperm structures and promotion of tyrosine phosphorylation of sperm proteins (Figure 3) [87–89]. 

To investigate the importance of protein tyrosine phosphorylation during epididymal maturation, most analyses were performed with the antiphosphotyrosine antibody. Using western blotting, the contents of plasma membranes were compared between hamster caput and cauda epididymal sperm, resulting in a differential phosphorylation pattern: the proteins with sizes of 94, 52, and 47 kDa looked more intense in cauda epididymal sperm while the 67 kDa band had more intensity in caput epididymal sperm [90]. However, western blotting detected caput epididymal sperm-specific phosphotyrosine expression in 93, 66, and 45 kDa bands in boar [91]; in addition, rat sperm from caput epididymis tended to show a stronger total band pattern of tyrosine phosphorylation than that of cauda epididymal sperm [37]. Immunofluorescence analyses with the antiphosphotyrosine antibody were performed to visualize the distribution of tyrosine phosphorylation in sperm. After permeabilization with methanol, boar sperm from proximal caput epididymis had strong labeling on the midacrosome as well as a faint signal on the whole tail. After transit through distal caput and corpus epididymides, this signal was detected only as a triangular shape on the posterior region of the midacrosome [91]. In mouse and rat, caput epididymal sperm, permeabilized with Nonidet P-40, resulted in fluorescence over the whole equatorial segment; however, the signal became restricted to a small region in the posterior equatorial segment after spermmoved to the cauda epididymis [92]. It is suggested that the equatorial segment plasma membrane works as a site of fusion with an oocyte membrane during fertilization; therefore, the accumulation of tyrosine phosphorylation may be connected to the later fusion process.

Lewis and Aitken have investigated the tyrosine phosphorylation pattern of sperm proteins after stimulation with cAMP by adding dibutyryl cAMP (db-cAMP) and pentoxifylline (PTX) [37]. By western blotting with the antiphosphotyrosine antibody in rat, the increase of cAMP resulted in more intense tyrosine phosphorylation bands in caput epididymal sperm proteins and much more intensity for cauda epididymal sperm proteins. However, this induction of tyrosine phosphorylation was inhibited by a protein kinase A (PKA)-inhibitor, H89 [37]. Immunofluorescence using the sperm fixed with methanol increased the signal in the tail region after db-cAMP/PTX stimulation [37]. Similar results were observed when the reduced form of NADPH (nicotinamide adenine dinucleotide phosphate) was added instead of db-cAMP, suggesting that this cAMP-dependent tyrosine phosphorylation is regulated by the redox system during epididymal maturation [93]. Furthermore, db-cAMP/PTX stimulation showed drastic change of the phosphotyrosine pattern in mouse sperm permeabilized with Triton X-100 as follows: staining on the acrosome and the principal piece of sperm from the proximal caput epididymis, strong on the midpiece as well as the acrosome and the principal piece of sperm from the distal caput and corpus epididymides, still strong on the midpiece and weak on the principal piece without any signal on the acrosome of sperm from the cauda epididymis [94]. It is also suggested that the signal leading to tyrosine phosphorylation in mouse sperm is negatively regulated by Ca^2+^ [95]. However, this inhibitory effect did not work when sperm arrived at the cauda epididymis [94]. Even with these observations, the mechanism of activation for this tyrosine phosphorylation has not been elucidated. One explanation of tyrosine phosphorylation in the midpiece is that the generation of reactive oxygen species (ROS) activates tyrosine phosphorylation signaling; however, the role of oxidative phosphorylation in sperm mitochondria is still controversial. At present, the role of tyrosine phosphorylation in the acrosome is unknown.

 The progress of proteomic analysis has contributed to the identification of sperm proteins that are important for epididymal maturation, including the protein phosphorylation process. Using two-dimensional fluorescence difference gel electrophoresis, eight rat sperm proteins were identified as candidates that undergo posttranslational modifications during epididymal maturation, and one of them, *β*-subunit of mitochondrial F_1_-ATPase, was serine-phosphorylated [96]. Recently, new methods using titanium dioxide have been developed to identify phosphopeptides, suggesting that 77 titanium-dioxide-enriched peptides (corresponding to 53 proteins) showed significant modifications during rat epididymal maturation [97].

 Here, if we focus on PTKs in epididymal epithelium, the receptor tyrosine kinase Ros and Src homology-2 (SH2-) domain-containing protein tyrosine phosphatase SHP-1 are expressed there. The mutant mice for Ros or for SHP-1 showed defects in the differentiation of the epididymis [98, 99]. Moreover, the sperm interact with various secretory proteins from epithelial cells of epididymides during epididymal transit, and some of them are proposed to be involved in sperm maturation [100, 101]. Therefore, it will be necessary to study the epididymal luminal environment as well as sperm proteins to obtain a deeper understanding of the role of PTKs in the sperm maturation process.

**Note**

During the processes of publishing the preent paper, one paper about Src and epididymal development and sperm functions was published [212]. As highlighted in our paper, Src has been identified as a PTK involved in capacitation-associated tyrosine phosphorylation downstream of PKA pathway. Added to this aspect, in this newly published paper, Visconti and colleagues reported that details about the male reproductive phenotypes of Src (KO) mice and Src localization in epididymis as well as in sperm. Src is not detected in caput epididymal sperm but in the midpiece and the postacrosomal region of cauda epididymal sperm. Src is also detected strongly in clear cells and weakly in principle cells of cauda epididymis and is shown to transfer into cauda epididymal sperm via epididymosomes during epididymal transit. Src KO mice have smaller size of cauda epididymis and reduced sperm motility, leading to unsuccessful in vitro fertilization.

## 5. Involvement of PTKs in Capacitation

**Figure 4:**
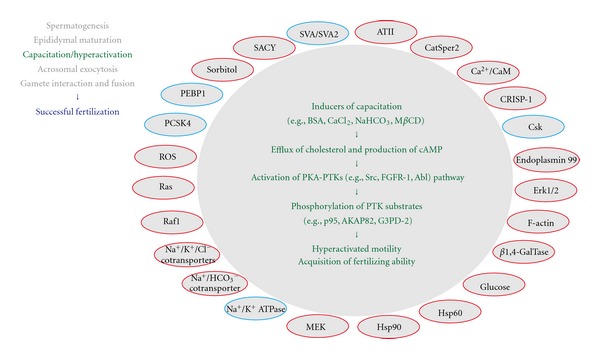
Protein-tyrosine phosphorylation and a sequence of events associated with capacitation and/or hyperactivation of sperm. For details of the capacitation/hyperactivation and its associated signaling molecules (the full spelling of all abbreviations as well), see text. Note that positive regulators for protein-tyrosine phosphorylation are indicated in red circles, whereas the negative regulators are indicated in blue circles.

Extratesticular sperm that has completed epididymal maturation must undergo a process called capacitation, a prerequisite for hyperactivated motility and acrosome reaction, in the female reproductive tract. Two researchers discovered this process independently in the 1950s [38, 39]. Later studies have demonstrated that capacitation can be reconstituted in vitro by using cauda (but not caput) epididymal or ejaculated sperm and artificial media supplemented with components that promote changes associated with in vivo capacitation. Capacitation seems to be a phenomenon specific to mammals, and accumulating evidence indicates that it generally involves a burst of protein-tyrosine phosphorylation (Figure 4).

 In mice, treatment of sperm with capacitation-inducing media promotes cAMP-dependent (i.e., PKA-dependent) tyrosine phosphorylation of several sperm proteins with molecular sizes of 116, 105, 95, 86, 76, and 54 kDa [102]. In particular, it is suggested that the 95 kDa phosphotyrosine-containing protein is identical to one that has been identified as a ZP3-dependent PTK substrate, namely, p95/zona receptor kinase (ZRK)/hexokinase (see below). Further studies by Visconti and colleagues have demonstrated that the sperm media should include bovine serum albumin (BSA), CaCl_2_, and NaHCO_3_ to induce capacitation and its associated tyrosine phosphorylation (proteins of 40–120 kDa) [103]. Interestingly, caput epididymal sperm, which lack an ability to undergo capacitation in vivo, cannot induce the tyrosine phosphorylation event in response to the treatment with capacitation media, indicating that epididymal maturation is required for the sperm response. In addition, it has also been shown that the requirement for BSA, CaCl_2_, and NaHCO_3_ in capacitation and associated PTK signaling is completely overcome by the addition of cAMP or its active analogs and that chemical inhibitors for PKAs (H-89, a substance that blocks ATP binding, and Rp-cAMPS, a nonhydrolysable AMP analog) interfere with the aforementioned processes [104]. These results clearly demonstrate that capacitation involves sequential activation of cAMP production and PKA-PTK pathway in response to the capacitation-inducing substances. 

 A similar system has also been demonstrated in other species including human [105] and mice of both domestic and wild-field species [106, 107]. Unlike mouse sperm, however, human sperm do not contain the 95 kDa phosphotyrosine-containing protein (p95/ZRK/hexokinase). Instead, the fibrous sheath proteins, AKAP82 (A-kinase/PKA anchoring protein 82: now referred as AKAP4), its precursor pro-AKAP82, and FSP95, a structural homolog of AKAP82, have been identified as prominently tyrosine-phosphorylated proteins in the capacitated sperm [108, 109]. Artificial Ca^2+^ signals, which promote the occurrence of acrosome reaction, lead to dephosphorylation of a subset of these phosphotyrosine-containing proteins. AKAP82 has also been identified as the major protein of the fibrous sheath of the mouse sperm flagellum, and its possible function to compartmentalize inactive PKA (before capacitation) to the cytoskeleton has been suggested [110]. Immunocytochemical and/or biochemical experiments also demonstrate that the tyrosine-phosphorylated forms of c-Abl tyrosine kinase are present in the capacitated human sperm [111]. 

 It has been shown that the cholesterol-binding heptasaccharides, methyl-*β*-cyclodextrin (M*β*CD) and OH-propyl-*β*-cyclodextrin, primarily promote release of cholesterol from the sperm plasma membrane and induce PTK signaling and capacitation in the absence of BSA [112, 113]. The M*β*CD's effects, like the BSA's effects, depend on both NaHCO_3_ and PKA activity, suggesting that they resemble those under physiological capacitation. In fact, BSA has also been shown to promote the release of cholesterol, and the addition of exogenous cholesterol interferes with the BSA-induced PTK signaling and capacitation [112]. These and other results suggest that efflux of cholesterol plays a pivotal role in upregulation of the cAMP-PKA-PTK pathway leading to capacitation [114]. 

 Other important factors that promote or suppress the onset of PTK signaling and/or capacitation include calmodulin, which may act as a positive regulator for the production of cAMP [115, 116]; seminal vesicle autoantigen, which has been shown to block BSA-induced capacitation [117, 118]; fertilization-promoting peptide or adenosine, which stimulates and inhibits PTK signaling in uncapacitated and capacitated sperm, respectively [119, 120]; extracellular glucose, whose shortage has been shown to delay the appearance of protein tyrosine phosphorylation [121]; Na^+^/HCO_3_^−^ cotransporter in the sperm, which provides Na^+^ ions as a positive regulator of PTK signaling and capacitation [122]; F-actin, whose generation and breakdown is required for capacitation and AE, respectively [123, 124]; endogenous redox activity, which is up-regulated under the control of the actions of HCO_3_^−^ [125, 126]; molecular chaperones such as hsp90, endoplasmin 99, and hsp60, which become tyrosine-phosphorylated upon capacitation and some of them may be involved in sperm-ZP recognition (in mouse, but not human) [127–129]; sperm-specific voltage-gated cation channel, CatSper2, whose gene knockout significantly alters sperm production; PTK signaling, which is associated with capacitation and induction of the AE [130]; extracellular Ca^2+^ ions, which may suppress tyrosine phosphorylation by decreasing the availability of intracellular ATP [131]; *β* 1,4-galactosyltransferase I, a possible ZP3-interacting protein whose gene knockout leads to precocious capacitation, which may be involved because of spontaneous elevation of cAMP [132]; angiotensin II, which is found in seminal plasma and has been shown to induce PTK signaling and capacitation via stimulation of adenylyl cyclase-dependent accumulation of cAMP [133]; HCO_3_^−^- and Ca^2+^-responsive soluble adenylyl cyclase (SACY), which has been identified as the dominant source of cAMP production [134, 135]; phosphatidylethanolamine-binding protein 1, a possible decapacitation factor, whose acquisition on the sperm surface during epididymal maturation and release before the onset of capacitation have been identified [136]; Na^+^/K^+^ ATPase, whose interaction with ouabain, a specific inhibitor of Na^+^/K^+^ ATPase, promotes PTK signaling and capacitation [137]; a 130 kDa CCCTC-binding nuclear factor, which becomes tyrosine-phosphorylated at capacitation and more potently binds to its target DNA sequence [138]; seminal vesicle protein secretion 2, which acts as a decapacitation factor by interacting with ejaculated sperm heads after copulation [139]; the cystic fibrosis transmembrane conductance regulator, a Cl^−^ channel that controls the activity of several transport proteins, including ENaCs (epithelial Na^+^ channels), and whose pharmacological inhibition leads to the failure of capacitation without affecting PTK signaling [34]; sorbitol, which is present in semen and has been shown to be effective in inducing PTK signaling via the action of sorbitol dehydrogenase [140]; Na^+^/K^+^/Cl^−^cotransporters, which may act as a source of chloride ions necessary for the onset of PKA-dependent PTK signaling at capacitation [141]; glycerol-3-phosphate dehydrogenase 2 (G3PD-2), which is expressed in the acrosome and principal piece and becomes tyrosine-phosphorylated upon capacitation [142, 143]; proprotein convertase subtilisin/kexin type 4 (PCSK4), whose null (thus impaired in fertility) sperm exhibit enhanced tyrosine phosphorylation in response to capacitation [144]; so-called Erk module, including Ras, Raf1, MAPK kinase (MAPKK/MEK), and Erk/MAPK, which is suggested to be involved in the presentation of phosphotyrosine-containing proteins on the sperm surface at capacitation [145].

Recent proteomics analysis has revealed more identities of tyrosine-phosphorylated proteins in response to capacitation: they include voltage-dependent anion channel, tubulin, pyruvate dehydrogenase E1 *β* chain, glutathione-*S*-transferase, NADH dehydrogenase (ubiquinone) Fe-S protein 6, acrosin-binding protein precursor (sp32), proteasome subunit *α*type 6b, and cytochrome b-c1 complex [42], although their functions remain to be elucidated. A more recent study has shown that Toll-like receptors 2 and 4 present on cumulus cells were activated by coculture with sperm in a hyaluronan fragment-dependent manner and that chemokines secreted from cumulus-oocyte complexes induced sperm PTK signaling and capacitation [146], providing evidence for the association of chemotaxis with capacitation. In addition, study of the knockout mouse suggests that an epididymal secretory protein CRISP-1 contributes to PTK signaling during capacitation [147].

Candidate PTKs related to capacitation include Src, whose interaction with PKA and enzymatic activation are seen in capacitated sperm [148, 149]; C-terminal Src kinase (Csk), whose negative regulatory function toward Src is canceled by serine phosphorylation (maybe by PKA) at capacitation [148, 150]; fibroblast growth factor receptor-1, whose dominant-negative mutant leads to the failure of PTK signaling and capacitation [151]; Abl tyrosine kinase, which is activated in response to capacitation in a PKA-dependent manner [152].

## 6. Involvement of PTKs in Acrosomal Exocytosis

**Figure 5:**
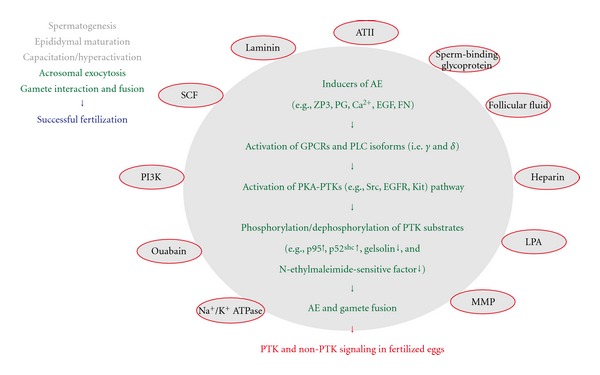
Protein-tyrosine phosphorylation and a sequence of events associated with acrosomal exocytosis. For details of the acrosomal exocytosis and its associated signaling molecules (the full spelling of all abbreviations as well), see text.

Early reports by Saling and colleagues have demonstrated that a 95 kDa mouse sperm protein, termed p95/ZRK (for zona receptor kinase)/hexokinase, is a tyrosine kinase substrate, whose phosphorylation level is elevated in response to sperm binding to zona pellucida glycoprotein ZP3 [153, 154]. It has long been believed that the physiological trigger for sperm AE is the binding of sperm to the ZP structures, namely, ZP3; so the aforementioned study has opened a window to analyze the roles of PTKs for AE. Subsequent studies have examined not only the physiological importance of the ZP3-induced tyrosine phosphorylation of the 95 kDa protein [155, 156] and other sperm proteins (e.g., 51 and 14–18 kDa proteins), but also the effect of several kinds of AE inducers other than ZP3 and/or various PTK or protein kinase inhibitors that could affect the tyrosine phosphorylation events associated with AE (Figure 5) [157–160]. A PTK inhibitor, tyrphostin, blocks the ZP-induced activation of PLC [161], suggesting that the *γ* isoform of PLC is involved in the ZP-induced PTK signaling leading to AE. A more recent study has shown, however, that *δ*4-isoform of PLC is essential for ZP3- or progesterone (PG)-induced Ca^2+^ release during mouse AE [162, 163]. AE induced by mannose-bovine serum albumin and an antibody against p95/ZRK, but not that induced by Ca^2+^ ionophore, can be inhibited by wortmannin, a specific inhibitor of PI3K, without inhibiting PTK signaling, implying a role of PI3K downstream of PTK signaling [164, 165]. AE induced by PG or platelet-activating factor has also been shown to involve an increase in protein-tyrosine phosphorylation of 75 and 97 kDa proteins, and PTK inhibitors (erbstatin, genistein) interfere with the induction of AE [166]. The PG-induced tyrosine phosphorylation is involved in the generation of the plateau phase of Ca^2+^ influx [167] and modulation of sperm GABAA-like receptor/chloride channel (chloride efflux) [168]. Study of domestic cat sperm has shown that ZP-induced AE, but not Ca^2+^ ionophore- or spontaneously induced AE, is inhibited by PTK inhibitors (genistein, tyrphostin), indicating that PTK signaling acts upstream of the Ca^2+^ increase during AE [169]. The involvement of PTK signaling mediated by Src has also been suggested for the promotion of capacitative Ca^2+^ entry, as reconstituted by thapsigargin treatment of sperm, during AE [170]. 

Tyrosine-phosphorylated proteins during AE also include p52^shc^, an isoform of the Shc adaptor proteins [171], a 107 kDa protein, whose phospholevel correlates well with the extent of AE (induced by Ca^2+^ ionophore) [172], and a heparin-binding sperm membrane protein (during AE induced by heparin) [173]. On the other hand, recent studies suggest the importance of proteintyrosine dephosphorylation in AE [174]. In support of this, tyrosine dephosphorylation of *N*-ethylmaleimide-sensitive factor, which undergoes SNARE complex disassembly, by protein-tyrosine phosphatase 1B has been shown to be required for the Ca^2+^ ionophore-induced AE [175], and gelsolin, an actin-severing protein that becomes tyrosinephosphorylated and inactivated during capacitation, has been shown to be dephosphorylated during AE, allowing its activation leading to actin depolymerization [176].

Another line of evidence demonstrates the identity of PTKs working during AE. An early report by Lax et al. showed that epidermal growth factor (EGF) can induce AE in bovine sperm [177], suggesting that EGF receptor (EGFR)/kinase is involved in this process. Further studies using this species have demonstrated that AE primarily involving activation of G protein-coupled receptors by lysophosphatidic acid or angiotensin II or AE induced by ouabain-Na^+^/K^+^ ATPase system promotes transactivation of EGFR/kinase via PKA-Src-matrixmetalloproteinase (MMP) or PKA-Src pathway [178, 179]. In the former system, G-protein-mediated production of AMP promotes PKA activation, PKA up-regulates Src (as seen in capacitated sperm), Src activates the secretion of heparin-binding EGF-like growth factor via MMP activation, thereby activating EGFR/kinase, and Src also affects the activity of EGFR/kinase through direct phosphorylation on tyrosine 845 [178], an Src-dependent phosphorylation site, whose phosphorylation has been implicated in some types of cancer cells [180–182]. Src activation and its importance for AE have also been demonstrated in humans [183, 184]. Another line of evidence suggests that SCF is involved in the promotion of mouse sperm AE through the activation of its cognate receptor/PTK c-Kit, PLC*γ*1, and phosphatidylinositol 3-kinase (PI3K) [185].

While many studies using mammals have shown the importance of PTK signaling in sperm AE, only limited findings have been described on the same subject in nonmammalian species. One potentially interesting finding reported recently demonstrates that egg components are capable of promoting protein-tyrosine phosphorylation and capacitation-like changes in sperm of the amphibian *Bufo arenarum* [186], implying its subsequent functions in AE.

 A recent report by Hirohashi and colleagues has shown that most fertilizing mouse sperm have undergone AE before contact with ZP during in vitro fertilization [46]. Furthermore, it has been shown that sperm binding to the zona pellucida is not sufficient to induce AE and that some mechanical process is important for physiological AE [187]. These results lead us to reconsider where and how sperm AE is initiated under physiological conditions and when and how PTK signaling contributes to the “real” AE. As described above, not only ZP3, but also other reagents or experimental conditions (e.g., PG) are reportedly inducible for AE in vitro. Additionally, possible oviductal substances such as sperm-binding glycoprotein [188], laminin [184], fibronectin [189], and follicular fluid [190] have been shown to induce AE accompanied by PTK signaling. Taking these findings together, further analysis focusing on the roles of the sperm microenvironment during capacitation and AE (before reaching the egg plasma membrane) in vivo should enable greater understanding of the physiological impact of PTK signaling. 

## 7. Involvement of PTKs in Gamete Interaction Fusion

Compared with the aforementioned categories of sperm biology, the relationship between sperm's PTK signaling and gamete interaction, especially at the level of plasma membranes (i.e., adhesion and fusion of gametes), has not yet been fully investigated. Immunocytochemical study demonstrates that sperm tail displays a time-dependent increase in tyrosine phosphorylation in response to ZP-free oocyte-sperm interactions [121], although its physiological importance and molecular detail have not yet been described. This seems to be mainly due to a technical problem in analyzing sperm functions at this point. In physiological conditions, a fertilizing spermatozoon closely interacts with or fuses with the plasma membrane of an egg, which has a protein content several hundredfold or more than that of a single sperm, so that the biochemical and cell biological experiments for evaluating not only protein tyrosine phosphorylation but also other molecular events associated with gamete interaction tend to fall into the analysis of those of the “fertilized egg (mixture of egg and sperm)” or egg itself, but not sperm itself. Under these circumstances, eggs of some animal species have been analyzed for the sperm or sperm-mimetic-induced PTK signaling. Accumulating evidence demonstrates that egg-associated Src and/or some other SFKs (i.e., Fyn and Yes) may play a crucial role for some events at fertilization: they include transient increase(s) in [Ca^2+^]_i_ concentrations (sea urchin, starfish, ascidian, fish, and frog) [191–194], MII spindle structures and functions (mammals) [195], and cleavage furrow ingression during mitosis (mammals) [196]. Roles played by the SFKs vary among species; however, it is worth noting that a wide range of animal species (from sea invertebrates to mammals) employ egg-associated SFKs as a sperm-induced trigger for activation of development. In this connection, it has been demonstrated that the sperm acrosomal or perinuclear theca-associated proteins may act as a trigger of signal transduction for initiation of development inside fertilized egg: so-called “sperm factors” (see “General View of Sperm Biology”). Among these are truncated c-Kit protein [197–199] and a WW domain-binding protein PAWP [200], both of which are specific proteins that may contribute to the modulation of the PTK signaling in eggs.

Egg analysis often involves parthenogenetic experiments, in which one or more of sperm's function-mimetic substances (e.g., Ca^2+^ ionophore) are used to reconstitute signaling events of fertilization, allowing easier functional evaluation of the egg-associated proteins. On the other hand, an absence of substitutes for sperm analysis has remained a problem. Ideally, some egg- or egg plasma membranemimetic substances, if applicable, would be helpful for solving this technical problem. In this regard, we suggest that egg membrane microdomains (MDs) could serve as excellent model materials of physiological value. As mentioned earlier, MDs or alternatively lipid/membrane “rafts” have been generally recognized as cholesterol-dependent micron- or nanometer-scaled membrane structures of cells, where a specific subset of glycosphingolipids, membrane-spanning and cytoplasmic proteins, and some other membrane components are assembled [201, 202]. Detailed analysis of egg MDs and fertilization signaling was first reported in sea urchin [203] and frog [204, 205], and thereafter, eggs or early embryos of mouse have also been documented to some extent [206, 207]. 

 In *Xenopus laevis*, the egg MDs are suggested to serve as a platform for sperm-induced Src PTK signaling. Namely, Src has been shown to be concentrated in the MDs of unfertilized eggs, and it is activated upon fertilization. M*β*CD treatment impairs the ability of eggs to undergo sperm induced initiation of development [204]. An MD-associated, transmembrane protein, uroplakin III, has been identified as a target of sperm protease, whose activity is required for *Xenopus* egg fertilization [208, 209], and as an intracellular substrate of Src [210]. In addition, we have found that sperm and some other sperm mimetics are capable of activating Src in MD fractions isolated from unfertilized *Xenopus* eggs, in vitro [211]. These results demonstrate that egg MDs would be useful materials for reconstitution of sperm-induced PTK signaling in the fertilized egg. If so, an opposite idea might also be valid, that is, egg MDs would be useful for reconstitution of egg (plasma membrane)-induced PTK signaling (or any other signaling event if it occurs) in the fertilizing sperm. To develop these ideas, we are now in the process of evaluating sperm functions before and after interaction with isolated egg MDs. It seems that this kind of reconstitution experiment can also be carried out in other animal species where isolation of egg MDs is possible, and thus its validity and physiological importance will soon be evaluated.

## 8. Conclusion and Perspectives

Among all the cells constituting multicellular organisms, egg and sperm are unique in terms of their history of production (i.e., gametogenesis, maturation, and/or differentiation), final structures, and physiological functions. In spite of enormous research efforts in recent years, many questions remain about how egg and sperm are produced and how they acquire their gamete-specific functions; in addition, new questions are continuously arising. Recent studies using pluripotent stem cells (e.g., embryonic or induced pluripotent stem cells) and/or molecular genetic approaches (e.g., gene knockout/KO and transgenic animals) have begun to disclose the genetic as well as cell biological background of gametogenesis, fertilization, and subsequent early embryogenesis. Moreover, study on the gametogenesis and fertilization in nonanimal species (e.g., plants, algae), which is not highlighted in this paper, and that in animal species have begun to merge, enabling researchers to learn more about the general scheme of sexual reproduction. Taking this background into account, it is certain that study on the signal transduction system involving protein-tyrosine phosphorylation in egg, sperm, and fertilized egg/zygote/early embryo will continue to be at the cutting edge of this research field.

## Abbreviations

PTK: Protein-tyrosine kinase
PGC: Primordial germ cell
SSC: Spermatogonial stem cell
cAMP: Cyclic adenosine monophosphate
AE: Acrosomal exocytosis
ZP: Zona pellucida
[Ca^2+^]_i_: Intracellular calcium
IP_3_: Inositol trisphosphate
PLC: Phospholipase C
SFK: Src family protein-tyrosine kinase
SCF: Stem cell factor
GDNF: Glial cell-derived neurotropic factor
BTB: Blood-testis barrier
FAK: Focal adhesion kinase
AJ: Adherence junction
ES: Ectoplasmic specialization
PI3K: Phosphatidylinositol 3-kinase
Erk: Extracellular signal-regulated kinase
MAPK: Mitogen-activated protein kinase
ATP: Adenosine trisphosphate
db-cAMP: Dibutyryl cAMP
PTX: Pentoxifylline
PKA: Protein kinase A
NADPH: Nicotinamide adenine dinucleotide phosphate
ROS: Reactive oxygen species
SH2: Src homology 2
SHP: SH2 domain-containing protein-tyrosine phosphatase
ZRK: Zona receptor kinase
BSA: Bovine serum albumin
AKAP: A-kinase/PKA-anchoring protein
M*β*CD: Methyl-*β*-cyclodextrin
SACY: Soluble adenylyl cyclase
ENaC: Epithelial Na^+^ channel
G3PD-2: Glycerol-3-phosphate dehydrogenase 2
PCSK4: Pro-protein convertase subtilisin/kexin type 4
MAPKK: MAPK kinase
Csk: C-terminal Src kinase
PG: Progesterone
EGF: Epidermal growth factor
MMP: Matrix metalloproteinase
MD: Membrane microdomain.
